# Risk of post-operative cardiovascular event in elderly patients with pre-existing cardiovascular disease who are undergoing hip fracture surgery

**DOI:** 10.1007/s00264-021-05227-7

**Published:** 2021-10-13

**Authors:** Yan Luo, Yu Jiang, Hongli Xu, Houchen Lyu, Licheng Zhang, Pengbin Yin, Peifu Tang

**Affiliations:** 1 Department of Orthopaedics, Chinese PLA General Hospital, Beijing, China; 2 National Clinical Research Centre for Orthopaedics, Sports Medicine and Rehabilitation, Beijing, China; 3 Medical Big Data Research Centre, Chinese PLA General Hospital, Beijing, China

**Keywords:** Pre-existing cardiovascular disease, Hip fracture, Post-operative cardiovascular events, Elderly

## Abstract

**Purpose:**

To evaluate the association between pre-existing cardiovascular disease (CVD) and the risk of developing post-operative cardiovascular event among elderly patients who underwent hip fracture surgery.

**Methods:**

We performed an observational study among patients with acute hip fracture aged at least 65 years and who received surgical intervention. Hip fracture patients with pre-existing CVD were matched for age, gender, fracture type, and year of admission with patients without pre-existing CVD. The primary endpoint was post-operative cardiovascular events, and patients were followed until discharge from hospital. Conditional logistic regression was used to determine the association between pre-existing CVD and post-operative cardiovascular event after adjusting for potential confounders including age, body mass index, time from fracture to surgery, pre-existing comorbidities, and the Charlson Comorbidity Index (CCI).

**Results:**

The study matched 858 pairs of patients with and without pre-existing CVD. Post-operative cardiovascular events developed in 40 and 14 patients with and without pre-existing CVD (44.6 versus 16.3 per 1000 persons), respectively. Compared to patients without pre-existing CVD, patients with any pre-existing CVD were more likely to develop post-operative cardiovascular events, with a crude odds ratio (OR) of 2.857 [95% confidence interval (CI), 1.554 to 5.251] and multivariable adjusted OR of 2.850 (95% CI, 1.318 to 7.139), respectively.

**Conclusion:**

In elderly patients who received hip fracture surgery, patients with pre-existing CVD are at a higher risk of developing post-operative cardiovascular events. Appropriate screening for this vulnerable population is recommended to prevent the risk of post-operative complications.

**Supplementary Information:**

The online version contains supplementary material available at 10.1007/s00264-021-05227-7.

## Introduction

Hip fracture occurs commonly among the elderly [1] with the number of hip fracture cases expected to rise from 1.6 million in 2000 to approximately 6.3 million in 2050 [2]. Most patients with hip fracture need surgical intervention. However, the incidence of post-operative complications in the hip fracture population is disproportionally high due to multiple pre-existing conditions. Post-operative cardiovascular events are frequent [3, 4] accounting for around 40% of all reported complications among patients receiving hip fracture surgery. Identifying patients with the greatest risk of developing post-operative cardiovascular events will help improve preoperative evaluation and inform clinical decision-making in hip fracture surgery.

Previous studies indicate that patients with pre-existing cardiovascular diseases (CVD) such as coronary heart disease, stroke, and heart failure are at a higher risk of developing long-term cardiovascular events. In the general population, patients with a history of CVD are at an increased risk for recurrent vascular events [myocardial infarction (MI), stroke, or vascular death] in a 17-year prospective cohort study [5]. Other researchers have also reported that Chinese patients who had any pre-existing MI or stroke were 2.87 times (Hazard ratio, HR = 2.872, 95% CI: 1.503–5.487) more likely to have a subsequent CVD event in 3 years [6]. In another study, for patients who received major surgical operations, peri-operative MI increased the risk of subsequent major adverse cardiac events (MACE) by 70% in the following year [7]. A study among hip fracture patients reported that patients with pre-existing heart failure had a higher one year post-operative heart failure exacerbation (HR = 3.00, 95% CI: 2.32–3.87) and one year mortality (HR = 2.11, 95% CI: 1.67–2.67) compared to those who did not have underlying or pre-existing heart conditions [8]. Of note, most reported studies have examined the association between pre-existing CVD and the long-term risk of developing cardiovascular events. Few studies have investigated the association between pre-existing CVD and post-operative cardiovascular events, which is a short-term risk following surgical intervention. Most patients with hip fractures are fragile with poor surgical tolerability. Pre-existing CVD combined with acute trauma as well as surgical stress could potentially increase the risk of post-operative cardiovascular events. The quantitative association between pre-existing CVD and post-operative cardiovascular events needs to be determined in this vulnerable population.

The aim of the study was to examine the association between common pre-existing CVD and post-operative cardiovascular events such as MI, stroke, and cardiovascular-related death among patients with hip fractures who have undergone surgery.

## Methods

### Data source and study population

We used data from the Chinese PLA General Hospital Hip Fracture Study (PLAGH Hip Fracture Study) [9], which is a single-centre cohort study in a tertiary hospital in Beijing, China. The study included all patients who were at least 65 years old with an admission diagnosis of femoral neck or intertrochanteric fracture between January 2000 and April 2016. Hip fractures were considered only if they were occurring for the first time and remedied by surgical intervention. Patients were excluded if they had a second hip fracture or if the fracture was not fresh, indicated by an admission more than three weeks after suffering the fracture.

### Study design and cohort definition

This was an observational cohort study intended to examine the association between pre-existing CVD and the risk of post-operative cardiovascular events among patients who received hip fracture surgery. Each patient with pre-existing CVD was matched for age (± 3 years), gender, fracture type, and year of admission to a hip fracture patient without pre-existing CVD.

Pre-existing CVD was defined as having one of the following diagnosis at admission: coronary heart disease (including MI, silent myocardial ischemia, angina pectoris), cerebrovascular events (including ischemic stroke and haemorrhagic stroke), peripheral artery disease and heart failure, and other cardiovascular diseases (including arrhythmia, valvular heart disease, and pulmonary heart disease) [10]. The diagnosis of the above conditions was undertaken by medical specialists (cardiologist or neurologist) based on patient history and medical records or admission results based on electrocardiogram, coronary computed tomography (CT), echocardiogram, and cranial CT/magnetic resonance imaging (MRI).

### Assessment of outcomes

The primary outcome was a composite endpoint of post-operative cardiovascular events, including post-operative myocardial infarction, stroke, and cardiovascular-related death [11, 12]. Secondary outcomes were (a) overall cardiovascular system-related events, including post-operative pulmonary embolism, angina pectoris, myocardial infarction, heart failure, arrhythmia, stroke, and cardiovascular-related death; (b) stroke; and (c) myocardial infarction. All diagnoses were confirmed by medical specialists (cardiologist, neurologist or internists from intensive care unit) based on post-operative lab tests, electrocardiogram, coronary or cranial CT, or MRI.

### Assessment of covariates

Demographic data included age, gender, and body mass index (BMI). The type of hip fracture was classified into either femoral neck fracture or intertrochanteric fracture according to the AO fracture classification [13]. Noted peri- and intra-operative factors include the type of surgical procedure (total hip replacement, semi-hip replacement, intramedullary nailing or other), type of anaesthesia (general anaesthesia, intra-spinal anaesthesia, nerve block and local anaesthesia), time from fracture to surgery, blood loss and transfusion, and operation duration time. Patients’ comorbidities were defined based on their medical history. Comorbidities of interest included type 2 diabetes, hypertension, chronic obstructive pulmonary disease (COPD), stroke sequelae (defined as the remaining language, mental, or physical dysfunction after stroke), dementia, and tumour. In addition, pre-existing disease burden was also calculated using the Charlson Comorbidity Index (CCI), which is a significant predictor of complications and both short-term and long-term mortality [14]. The CCI score was summed up according to specific weights assigned to each condition [15].

### Statistical analysis

For the comparison of baseline characteristics between patients with and without pre-existing CVD, we used Chi-square or Fisher’s exact test for categorical variables, row mean scores difference test for ordinal variables, and Mann–Whitney *U* test/ t-test for non-normal/normal continuous variables. A simple imputation method using the medium was used for a few missing values in blood loss and surgery time. We calculated the risk of post-operative cardiovascular events in both groups. The conditional logistic regression was used in the 1:1 matched dataset to evaluate the risk of developing post-operative cardiovascular events, adjusting for potential confounders (age, body mass index, time from fracture to surgery and type 2 diabetes, hypertension, COPD, stroke sequelae, dementia, tumour, and the CCI score). In a sensitivity analysis, we repeated the same analysis in the full cohort using unconditional logistic regression. To evaluate whether the pre-existing CVD can serve as a useful clinical characteristic to identify high-risk patients with regard to developing post-operative cardiovascular events, we performed an exploratory analysis by examining the proportion of recurrent cases (patients had a history of this condition) for arrhythmia, stroke, heart failure, and MI. All statistical analyses were performed using the R software (R Foundation, version 3.5.1), and *P* < 0.05 was considered statistically significant.

## Results

**Fig. 1:**
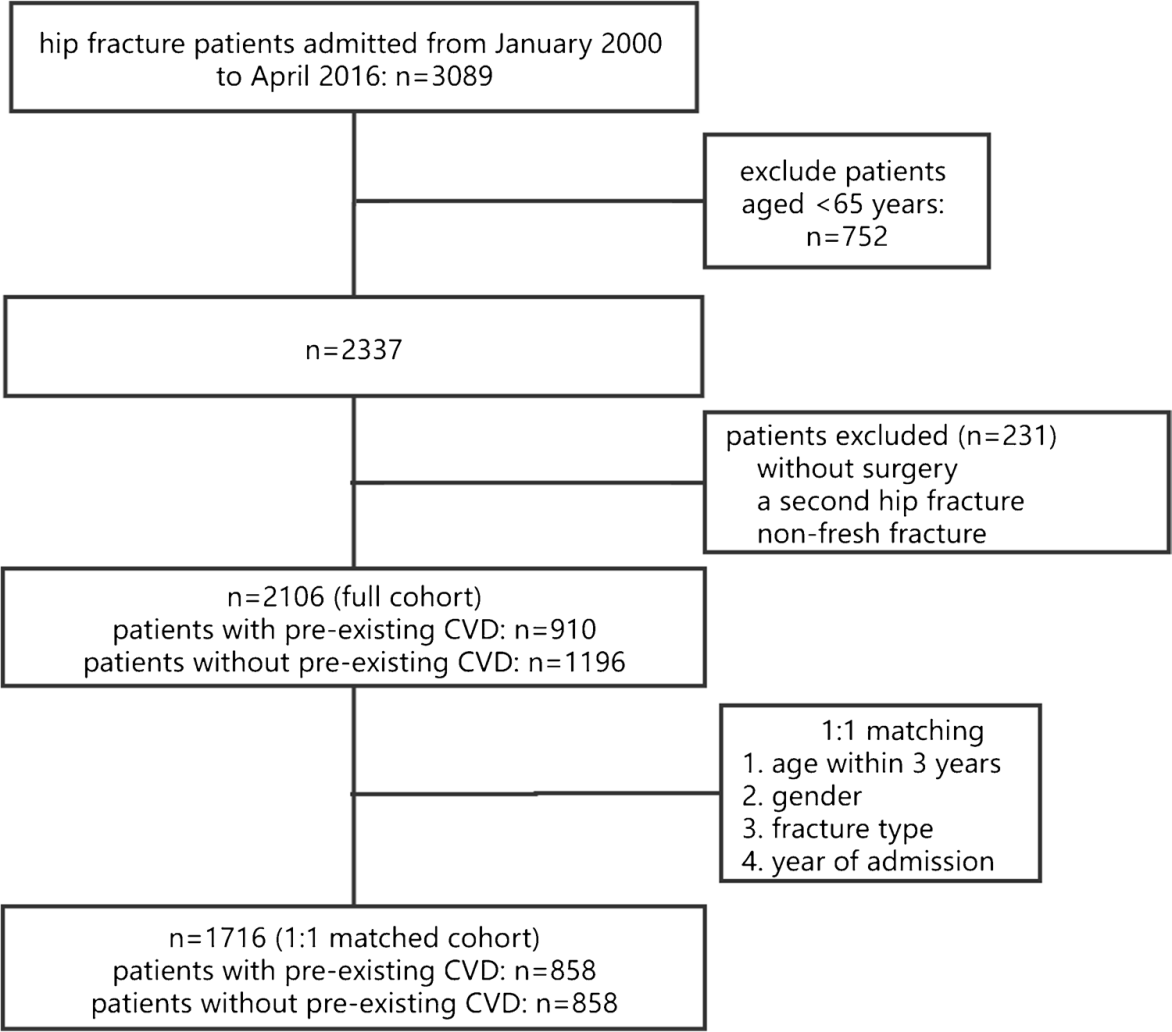
Study population. CVD, cardiovascular disease

Of the 3089 patients sampled between the study period, 2106 (68.2%) were included in this analysis. The medium age of the included patients was approximately 79.0 (interquartile range, IQR: 10.5) years, 67.5% of them were females, and 48.7% of them had femoral neck fracture. There were 910 of 2106 (43.2%) patients who had pre-existing CVD. After performing the (1:1) matching by age, gender, fracture type, and admission year, 858 patients (94.3%) with pre-existing CVD were matched to 858 patients without pre-existing CVD (Fig. 1). In the matched dataset, patients with pre-existing CVD had a higher burden of comorbidities such as type 2 diabetes (29.1% vs. 16.9%), hypertension (65.3% vs. 39.4%), and chronic kidney disease (4.3% vs. 2.0%) when compared to those without pre-existing CVD. The proportion of CCI index over 2 was 41.4% in patients with pre-existing CVD and 12.7% in those without pre-existing CVD (Table 1).

**Table 1:** Baseline characteristics of the study population in the matched cohort

	Patients without pre-existing CVD* (*n* = 858)	Patients with pre-existing CVD (*n* = 858)	*P*
*n*	858	858	
Age (median [IQR])	79.06 [74.12, 84.12]	79.37 [74.62, 83.85]	0.849
BMI (median [IQR])	22.49 [19.78, 24.44]	22.66 [20.76, 25.09]	< 0.001
Gender (%)			1.000
Male (%)	289 (33.7)	289 (33.7)	
Female (%)	569 (66.3)	569 (66.3)	
Fracture type (%)			1.000
Intertrochanteric fracture (%)	434 (50.6)	434 (50.6)	
Femoral neck fracture (%)	424(49.4)	424(49.4)	
Surgery type (%)			0.410
Total hip replacement	70 (8.2)	57 (6.6)	
Semi-hip replacement	372 (43.4)	391 (45.6)	
Intramedullary nailing	405 (47.2)	394 (45.9)	
Other^#^	11 (1.3)	16 (1.9)	
Anaesthesia (%)			0.243
General anaesthesia	285 (33.2)	258 (30.1)	
Intra-spinal anaesthesia	287 (33.4)	276 (32.2)	
Nerve block	278 (32.4)	317 (36.9)	
Local anaesthesia	8 (0.9)	7 (0.8)	
Time from fracture to surgery (day) (median [IQR])	6.00 [4.00, 10.00]	7.00 [4.00, 11.25]	< 0.001
Blood loss in surgery (mL) (median [IQR])	200.00 [100.00, 300.00]	200.00 [100.00, 300.00]	0.714
Surgery time (min) (median [IQR])	105.00 [84.50, 133.50]	105.00 [85.00, 135.00]	0.526
Blood transfusion (%)	214 (27.8%)	232 (29.5%)	0.486
Comorbidities			
Type 2 diabetes (%)	145 (16.9)	250 (29.1)	< 0.001
Hypertension (%)	338 (39.4)	560 (65.3)	< 0.001
Myocardial Infraction (%)	-	95 (11.1)	-
Coronary heart disease (%)	-	413 (48.1)	-
Arrhythmia (%)	-	225 (26.2)	-
Heart failure (%)	-	26 ( 3.0)	-
Stroke (%)	-	389 (42.7)	-
Stroke sequelae (%)	-	97 (10.7)	-
COPD (%)	53 ( 6.2)	63 ( 7.3)	0.387
Pneumonia within 3 months (%)	21 ( 2.4)	31 ( 3.6)	0.205
Dementia (%)	48 ( 5.6)	42 ( 4.9)	0.588
Tumour (%)	63 ( 7.3)	71 ( 8.3)	0.529
Malignant tumour (%)	39 ( 4.5)	49 ( 5.7)	0.325
Rheumatic disease (%)	13 ( 1.5)	19 ( 2.2)	0.372
CKD (%)	17 ( 2.0)	37 ( 4.3)	0.009
Peptic ulcer disease (%)	63 ( 7.3)	80 ( 9.3)	0.162
Liver disease (%)	15 ( 1.7)	21 ( 2.4)	0.400
CCI (%)			< 0.001
0	535 (62.4)	196 (22.8)	
1	214 (24.9)	307 (35.8)	
1 +	109 (12.7)	355 (41.4)	
Pre-operative haemoglobin, Hb (g/L) (mean (SD))	38.78 (4.28)	38.97 (4.38)	0.383
Post-operative haemoglobin, Hb (g/L) (mean (SD))	32.45 (3.93)	32.87 (4.36)	0.046
Pre-operative serum creatinine, Cr (umol/L) (mean (SD))	75.43 (55.40)	79.04 (55.05)	0.178
Post-operative serum creatinine, Cr (umol/L) (mean (SD))	71.88 (57.85)	75.36 (54.80)	0.208
Pre-operative serum calcium, Ca (mmol/L) (mean (SD))	2.24 (0.15)	2.25 (0.14)	0.200
Post-operative serum calcium, Ca (mmol/L) (mean (SD))	2.13 (0.15)	2.15 (0.16)	0.056
Pre-operative serum albumin, Alb (g/L) (mean (SD))	38.78 (4.28)	38.97 (4.38)	0.383
Post-operative serum albumin, Alb (g/L) (mean (SD))	32.45 (3.93)	32.87 (4.36)	0.046
Pre-operative C-reactive protein, CRP (mg/dL) (mean (SD))	6.07 (5.65)	6.13 (6.11)	0.862
Post-operative C-reactive protein, CRP (mg/dL) (mean (SD))	5.67 (4.23)	5.38 (4.15)	0.195
Pre-operative D-dimer (μg/mL) (mean (SD))	6.86 (6.06)	7.13 (5.94)	0.377
Post-operative D-dimer (μg/mL) (mean (SD))	5.18 (4.67)	4.48 (4.58)	0.021

*CVD*, cardiovascular disease; *IQR*, interquartile range; *BMI*, body mass index; *COPD*, chronic obstructive pulmonary disease; *CKD*, chronic kidney disease; *CCI*, Charlson Comorbidity Index

^*^Pre-existing CVD: we defined a history of pre-existing CVD as having one of the following conditions: coronary heart disease (including myocardial infarction, silent myocardial ischemia, angina pectoris), cerebrovascular events (including ischemic stroke and haemorrhagic stroke), heart failure, and other cardiovascular diseases (including arrhythmia, valvular heart disease, and pulmonary heart disease)

^#^Other surgery types including extramedullary fixation, plate, external fixator, and simple fixation

For the primary endpoint, post-operative cardiovascular events occurred in nearly thrice as many patients with pre-existing CVD (40) as those without underlying CVD (14). Compared to patients without pre-existing CVD, the crude OR of developing post-operative cardiovascular events in patients with pre-existing CVD was 2.857 (95% CI: 1.554–5.251). After adjusting for potential confounders, the multivariable-adjusted OR was 2.850 (95% CI: 1.138–7.139). Similarly, for the secondary endpoints, there was a higher risk of developing overall cardiovascular system-related events among patients with pre-existing CVD (107) with a risk of 124.7 (95% CI, 102.6 to 146.8) per 1000 persons compared to those without previous CVD (Table 2).

**Table 2:** Risk of post-operative CVD events in patients with and without pre-existing CVD in the matched cohort

	Patients with pre-existing CVD			Patients without pre-existing CVD			Crude *OR* (95% *CI*)	Adjusted *OR* (95% *CI*)^a^
	*n*	No. of events	Risk per 1000 persons	*n*	No. of events	Risk per 1000 persons		
Primary endpoint								
Post-operative cardiovascular events^*^	858	40	46.6 (33.5–62.9)	858	14	16.3 (8.9–27.2)	2.857 (1.554–5.251)	2.850 (1.138–7.139)
Secondary endpoints								
Overall cardiovascular system-related events^#^	858	107	124.7 (102.6–146.8)	858	30	35.0 (23.7–49.5)	3.750 (2.472–5.690)	3.679 (2.115–6.399)
Stroke	858	32	37.3 (25.6–52.2)	858	8	9.3 (4.0–18.3)	4.000 (1.843–8.680)	5.618 (1.386–19.271)
Myocardial Infraction	858	6	7.0 (1.4–12.6)	858	4	4.7 (1.3–11.9)	1.500 (0.423–5.315)	-

*CVD*, cardiovascular diseases; *OR*, odds ratio; *CI*, confidence interval

^*^Post-operative cardiovascular events, including post-operative myocardial infarction, stroke, and cardiovascular-related death

^#^Overall cardiovascular system-related events, including post-operative pulmonary embolism, angina pectoris, myocardial infarction, heart failure, arrhythmia, stroke and cardiovascular-related death

^a^Adjusted factors: age, body mass index, time from fracture to surgery, and comorbidities including type 2 diabetes, hypertension, chronic obstructive pulmonary disease, stroke sequelae, dementia, tumour, and the Charlson Comorbidity Index

We performed the same analysis in the full cohort as a sensitivity evaluation. The results were consistent with the primary analysis for both the primary endpoint and three secondary endpoints. For example, compared to patients without pre-existing CVD, the crude and multivariable-adjusted OR for post-operative cardiovascular events were 3.440 (95% CI: 1.985–6.234) and 3.367 (95% CI: 1.783–6.566), respectively (Table 3).

**Table 3:** Sensitivity analysis for the risk of post-operative complications in patients with and without pre-existing CVD in the full cohort

	Patients with pre-existing CVD			Patients without pre-existing CVD			Crude *OR* (95% *CI*)	Adjusted *OR* (95% *CI*)^a^
	*n*	No. of events	Risk per 1000 persons	*n*	No. of events	Risk per 1000 persons		
Primary endpoint								
Post-operative cardiovascular events ^*^	910	43	47.3 (34.4–63.1)	1196	17	14.2 (8.3–22.7)	3.440 (1.985–6.234)	3.367 (1.783–6.566)
Secondary endpoints								
Overall cardiovascular system-related events^#^	910	113	124.2 (102.7–145.6)	1196	45	37.6 (27.6–50.0)	3.626 (2.556–5.229)	3.909 (2.613–5.920)
Stroke	910	34	37.4 (26.0–51.8)	1196	11	9.2 (4.6–16.4)	4.181 (2.176–8.691)	3.515 (1.657–7.920)
Myocardial Infraction	910	7	7.7 (3.1–15.8)	1196	4	3.3 (0.9–8.5)	2.310 (0.695–8.842)	2.917 (0.721–12.730)

*CVD*, cardiovascular diseases; *OR*, odds ratio; *CI*, confidence interval

^*^Post-operative cardiovascular events, including post-operative myocardial infarction, stroke, and cardiovascular-related death

^#^Overall cardiovascular system-related events, including post-operative pulmonary embolism, angina pectoris, myocardial infarction, heart failure, arrhythmia, stroke, and cardiovascular-related death

^a^Adjusted factors: age, gender, fracture type, year of admission, time from fracture to surgery, body mass index, and comorbidities including type 2 diabetes, hypertension, chronic obstructive pulmonary disease, stroke sequelae, dementia, tumour, and the Charlson Comorbidity Index

**Fig. 2:**
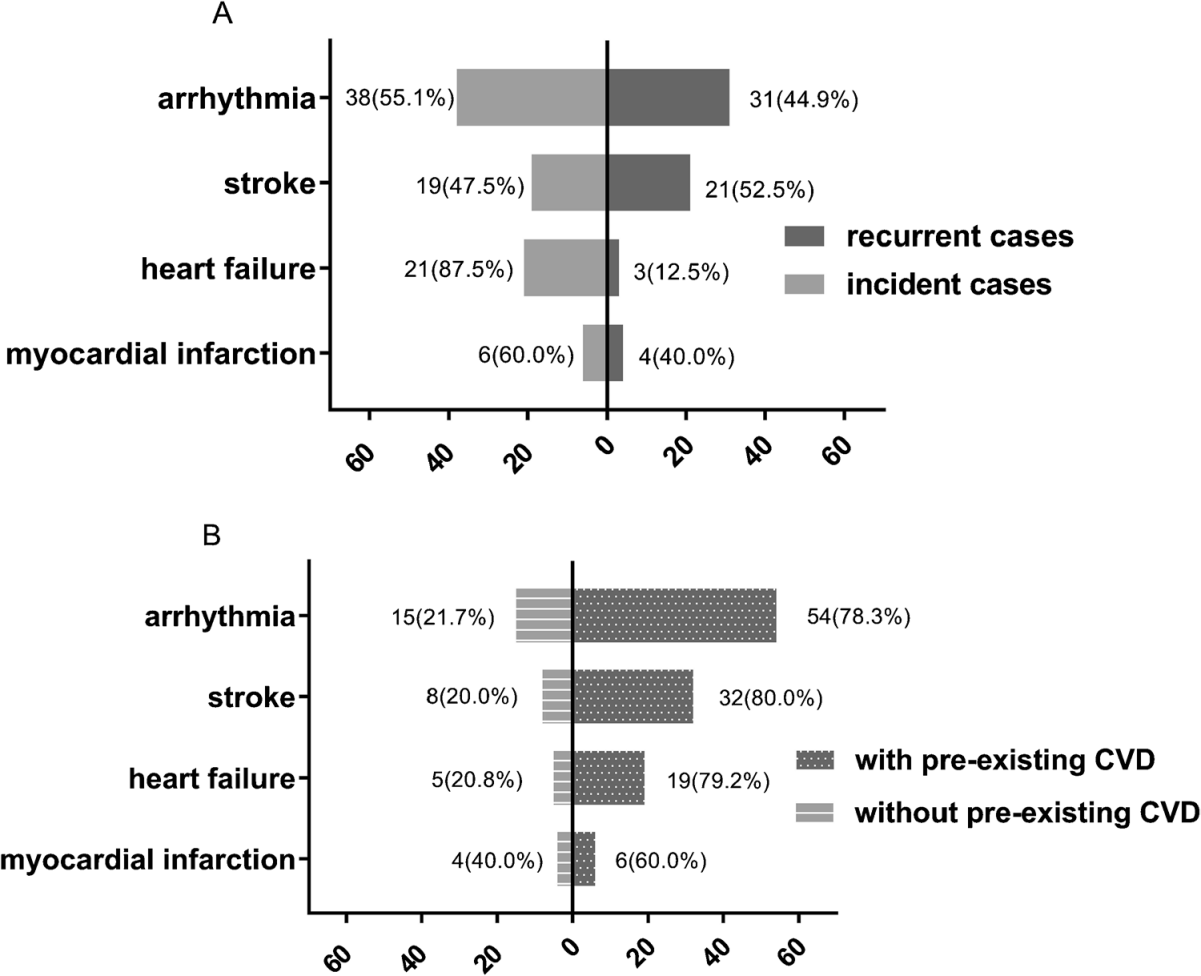
(**A**) Recurrent cases of post-operative arrhythmia, stroke, heart failure, and myocardial infarction. (**B**) Cases of pre-existing cardiovascular disease (CVD)

In an exploratory analysis, we examined the proportion of recurrent cases and new cases for post-operative arrhythmia, stroke, heart failure, and MI (Fig. 2). In the primary dataset of the 69 post-operative arrhythmia events, 31 (44.9%) of them had a history of arrhythmia (Fig. 2A), but 54 (78.3%) of them had a history of any pre-existing CVD as defined for this study (Fig. 2B). Of the 40 stroke events, 21 (52.5%) of them had a history of stroke, but 32 (80.0%) had a history of any pre-existing CVD. Of the 24 post-operative heart failure events, three (12.5%) had a history of heart failure, while 19 (79.2%) had a history of any pre-existing CVD. Similar results were found for post-operative MI (Fig. 2).

## Discussion

In this study, we examined the association between pre-existing CVD and the risk of developing post-operative cardiovascular events in geriatric patients receiving hip fracture surgery. Patients with pre-existing CVD showed an increased risk of post-operative cardiovascular events, including MI, stroke, and cardiovascular-related death, compared to patients without pre-existing CVD.

To the best of our knowledge, this is the first study to examine the association between pre-existing CVD and short-term post-operative cardiovascular events, among patients who have received hip fracture surgery. Such patients who have pre-existing CVD were 2.85 times more likely to develop post-operative cardiovascular events following hip fracture surgery. This result highlights that patients with pre-existing CVD are a vulnerable subgroup with a heightened risk of developing post-operative cardiovascular events.

In a previous study, Jorgensen and colleagues reported that patients with pre-existing stroke have an increased risk of major adverse cardiac events following elective non-cardiac surgery. In that study, the 30-day crude incidence of major adverse cardiac events following elective non-cardiac surgery was over ten times higher in patients with pre-existing stroke. Furthermore, patients with pre-existing stroke were four times more likely to develop post-operative major adverse cardiac events [16]. In patients receiving joint replacement surgery, a study using national inpatients sample (USA) showed that the history of cardiac diseases is a risk factor for acute myocardial infarction following surgery, with an OR of 4.9 for pre-existing coronary artery disease, 2.6 for congestive heart failure, and 1.2 for valvular disease [17].

Compared to patients receiving elective surgery, patients with hip fracture are more fragile and less tolerant to surgical stress. In view of the high incidence of post-operative cardiovascular events in hip fracture patients, identifying useful risk factors will help physicians find the most vulnerable subgroup of patients. In this study, we showed that pre-existing CVD is a strong risk factor for developing post-operative cardiovascular events.

The current study further evaluated whether pre-existing CVD could serve as a useful clinical characteristic to identify high-risk patients for developing post-operative cardiovascular events. Among patients who experienced post-operative cardiovascular events including MI, stroke, and cardiovascular-related death, the proportion of patients having pre-existing CVD is as high as 80%. For each specific post-operative cardiovascular event (MI, stroke, arrhythmia, and heart failure), most patients had at least one pre-existing CVD. Considering patients who developed MI, 60% of them had pre-existing CVD. For the other three specific categories, the proportions were even higher (Fig. 2B). By contrast, only half of these are recurrent cases for each specific event (Fig. 2A). This observation suggests that any type of pre-existing CVD is a strong risk factor for post-operative cardiovascular event and could possibly be useful in identifying patients at high risk of developing post-operative cardiovascular event.

This study not only highlights the fact that patients who have fractured hips with pre-existing CVD have increased risk of post-operative cardiovascular events, but also provides some quantitative aspects of the risks. The outcomes of this study promise to be useful to clinicians in providing insights during pre-operative evaluation of elderly patients with hip fractures who have CVD comorbidities.

Currently, there is no routine post-operative CVD events surveillance framework. Framingham Risk Score (FRS) [18] and Atherosclerotic Cardiovascular Disease (ASCVD) [19] risk assessment tools are useful risk scoring systems in predicting CVD risk among non-hip fracture populations. Therefore, the validity and usefulness of applying these scores in predicting post-operative cardiovascular events among patients with hip fractures are unclear. Expanding these predictive models to cater for patients with hip fractures by incorporating some population-specific risk factors is an unmet need. Recently, some potentially useful risk factors or biomarkers of post-surgical complications have been proposed by several studies. These risk factors include old age, poor physical status, and receiving pre-operative red blood cell transfusions frequently [1]. In addition, some biomarkers, such as levels of brain natriuretic peptide (NT-proBNP) [20, 21], troponin I [22, 23], and haemoglobin [24], have demonstrated their potential for assessing the risk of the occurrence of CVD events after surgery. In this study, we found that one or a combination of pre-existing CVD can serve as a simple and yet effective marker to identify patients vulnerable to post-operative cardiovascular events. Based on the study results, pre-existing CVD should be incorporated in models for predicting post-operative cardiovascular events in patients with hip fractures who go through hip surgery.

This study has some strengths. First, we used data from a large hip fracture cohort. Second, pre-existing CVD and post-operative cardiovascular events were well adjudicated. All these CVD-related diagnoses were made by specialists based on necessary lab tests, electrocardiogram, echocardiogram, and coronary CT/MRI. Third, we used robust study design to examine the association between pre-existing CVD and post-operative cardiovascular events in hip fracture patients. This analysis filled in the knowledge gap over the short-term risk of cardiovascular events after surgery and could be useful for decision marking. Meanwhile, this study has some limitations. Although a large study population was included, this study is a single-centre study. In the 1:1 matching process, 52 (5.7%) patients with pre-existing CVD were not matched with controls since we used strict matching criteria, which may lead to potential biases. However, a sensitivity analysis using all patients with pre-existing CVD did not change our estimates substantially.

## Conclusions

In elderly patients who have undergone hip fracture surgery, pre-existing CVD was a great risk factor for developing post-operative cardiovascular events. Nearly 80% of patients who experienced post-operative cardiovascular events had at least one pre-existing CVD. Appropriate screening strategies are needed for this vulnerable population to reduce the incidences of post-operative CVD-related complications.

## Supplementary Information

Below is the link to the electronic supplementary material.

Supplementary file1 (DOC 90 KB)

## Data Availability

The datasets used or analysed during the current study are available from the corresponding author on reasonable request.
